# Therapeutic Mechanisms of Exercise in Parkinson’s Disease

**DOI:** 10.3390/ijms26104860

**Published:** 2025-05-19

**Authors:** Alice C. Wilson, Dean L. Pountney, Tien K. Khoo

**Affiliations:** 1 School of Medicine and Dentistry, Griffith University, Gold Coast, QLD 4222, Australia; 2 School of Pharmacy and Medical Sciences, Griffith University, Gold Coast, QLD 4222, Australia; 3 Graduate School of Medicine, University of Wollongong, Wollongong, NSW 2500, Australia; 4 Northern New South Wales Local Health District, NSW Health, Lismore, NSW 2480, Australia

**Keywords:** Parkinson’s disease, exercise, management, mechanism

## Abstract

Despite being the second-most common neurodegenerative disease, the etiology of Parkinson’s disease (PD) remains uncertain with current knowledge suggestive of multiple risk factors. Furthermore, curative treatment does not yet exist, and treatment is primarily symptomatic in nature. For this reason, supportive therapies such as exercise are a crucial tool in PD management. It is useful to better understand how exercise affects the brain and body in the context of PD to guide clinical decision-making and determine the optimal exercise intensity and modality for PD patients. This review outlines the various mechanisms by which exercise can be beneficial as a therapeutic option in PD.

## 1. Introduction

Parkinson’s disease (PD) is the second-most common neurodegenerative disease, and its etiology remains to be elucidated. PD continues to be diagnosed primarily based on clinical criteria, which require the presence of bradykinesia along with supportive cardinal motor signs; however, a wide range of non-motor symptoms can also occur. The latter includes autonomic dysfunction, mood changes, cognitive impairment, and psychosis, which can be more frequent with disease progression [1]. The pathophysiological hallmark of PD involves loss of dopaminergic neurons in the substantia nigra pars compacta (SNc) and aggregation of misfolded alpha-synuclein (α-Syn) into Lewy body intraneuronal inclusions. The loss of dopaminergic neurons results in an imbalance between dopamine and other neurotransmitters such as glutamate, as well as other pathological cascades such as neuroinflammation and mitochondrial dysfunction [2,3]. It is becoming increasingly recognized that PD is a heterogeneous disease with a multifactorial etiology [4]. One hypothesis implicates the gut microbiome and disruption to the gut–brain axis, and many studies link PD with aging and environmental exposure. Mitochondrial dysfunction, oxidative stress, metal ion dyshomeostasis, neuroinflammation, compromise of the glymphatic system and several gene mutations have been shown to contribute to Parkinsonian etiology [1,5,6,7].

Due to the lack of curative potential of current existing PD treatment options, adjunctive therapies such as exercise are often recommended, and their benefits are increasingly supported in multiple studies [8,9,10]. Aside from improving general health with minimal negative consequences, exercise is thought to be neuroprotective and has been shown to play a therapeutic role in many neurodegenerative diseases that may delay both disease onset and progression [11,12]. In the context of PD, numerous mechanisms can explain the beneficial effects of exercise, and this will be further detailed in this review. Understanding these mechanisms can help justify the role of exercise in PD and direct further research into targeted physical therapies for those with debilitating disease who can no longer be physically active.

## 2. Neurotrophic Factors

Neurotrophic factors are important in neuronal health and in the context of neurodegeneration. They have a range of neuroprotective roles, including the promotion of neuron proliferation and survival, synapse formation and reduction of α-Syn aggregation [13,14,15,16]. There are three broad categories of neurotrophic factors implicated in PD; neurotrophins such as brain-derived neurotrophic factor (BDNF), glial cell line-derived neurotrophic factor (GDNF)-family ligands, and the cerebral dopamine neurotrophic factor/mesencephalic astrocyte-derived neurotrophic factor (CDNF/MANF) family [17]. BDNF and GDNF are the best characterized neurotrophic factors and produce similar effects in the brain [17]. The main mechanism of BDNF signaling is via tropomyosin-related kinase B (TrkB) receptors, which activate downstream signaling pathways that result in neuronal differentiation and survival, anti-apoptotic gene expression and Ca^2+^ mobilization for synaptic plasticity [18,19,20]. GDNF activates similar pathways but does so through the Rearranged During Transfection (RET) receptor [21,22]. More recently, the beneficial effects of CDNF and MANF signaling in PD have been investigated, and although these molecules are still classed as neurotrophic factors, they function quite differently to other biomolecules [15,17]. It has been suggested that the chronic endoplasmic reticulum (ER) stress response and the Unfolded Protein Response (UPR) are involved in PD pathogenesis [23]. CDNF and MANF both reside in the ER and downregulate the UPR pathway, which can promote dopaminergic neuron survival [15].

Given their potential for therapeutic use, trials of exogenous BDNF and GDNF administration have been undertaken; however, the outcomes have been inconsistent [13]. In contrast, exercise has been found to increase levels of endogenous neurotrophic factors in rodent models [24,25], and may do so regardless of disease severity [26]. This result is supported by increased levels of blood BDNF-TrkB signaling in PD patients after exercise [27]. A recent systematic review and meta-analysis concluded that the type of exercise has no significant impact on BDNF levels, but higher intensity exercise is likely to be a more effective and beneficial determinant [28]. This notion is also true for CDNF but not MANF [29]. In contrast, a study investigating GDNF levels in the spinal cord of healthy rats found that lower intensity, involuntary exercise increased GDNF more than other modalities [30]. The latter finding has not been corroborated by human studies.

## 3. Synaptic Regulation

Recent studies have demonstrated that physical activity can modulate synaptic plasticity in brain regions affected by PD, particularly glutamatergic inputs into the basal ganglia and dopaminergic neurotransmission within basal ganglia structures [17]. Following dopaminergic neuron loss, imbalances in glutamatergic and dopaminergic neurotransmission occur, which modify the activity of direct and indirect basal ganglia loops [31]. The striatum contributes to both direct and indirect loops via projections of medium spiny neurons (MSNs). Direct pathway MSNs express excitatory D1 dopamine receptors, and indirect pathway neurons express inhibitory D2 receptors [32]. Both direct and indirect MSNs have dendrites with dense dendritic spines, and receive a combination of dopaminergic inputs from the SNc and glutamatergic inputs from the cortex and thalamus [33]. Loss of SNc dopaminergic input into the striatum—a pathophysiological hallmark of PD—results in disruption to the homeostasis of dopamine and glutamate neurotransmission [17]. Reduced dopamine activation of D2 receptors on striatal MSNs leads to excessive glutamate in the synaptic cleft from either increased presynaptic release or impaired reuptake [2]. This increases calcium signaling through N-methyl-D-aspartate (NMDA) receptor activation or voltage-gated calcium channels, which can lead to calcium overload on MSN dendrites [34]. Consequently, the density, length and total number of MSN dendritic spines are reduced in PD [35].

It appears that exercise can remediate these pathological changes to some extent by reducing the amount of glutamate in the synaptic cleft and restoring dendritic spine density [36,37]. Some studies have also suggested that exercise could stimulate neurogenesis of substantia nigra or striatum dopaminergic neurons, or increase expression of the dopamine reuptake transporter to increase the availability of dopamine in presynaptic neurons [38,39]. For example, a small study by de Laat et al. [39] found that six months of treadmill exercise increased dopamine transporter availability and substantia nigra neuromelanin content on PET imaging in patients with mild PD. However, studies based on induced PD rodent models were unable to replicate similar findings [37,40]. Additionally, the effect of exercise on striatal MSN α-amino-3-hydroxy-5-methyl-4-isoxazolepropionic acid (AMPA) receptors can upregulate the GluA2 subunit of the receptor in rodent models, rendering it less permeable to calcium and thereby protecting the MSN from excitotoxicity [41,42]. Though it is currently unclear what the best intensity and modality of exercise for improving synaptic plasticity may be, several studies have found a positive correlation between high- or moderate-intensity aerobic exercise and measures of synaptic plasticity [39,43], but the effects of low-intensity or resistance exercise have not yet been investigated.

## 4. Neural Oscillation

Functional deficits in PD are associated with abnormalities in the oscillatory activity of basal ganglia neurons [44]. Degeneration of dopaminergic neurons causes an imbalance in excitatory and inhibitory input into the basal ganglia, leading to abnormal functioning of the cortico-basal ganglia-thalamo-cortical (CBGTC) loop [45,46]. The abnormal oscillatory activity arises from dysfunctional coordination [47]; specifically, the motor cortex and various basal ganglia nuclei have been studied regarding abnormal firing rates, excessive beta frequency oscillations and unusual neuronal synchronicity [48]. This has been supported by studies using animal and human PD models [45]. It remains contentious in the literature as to exactly how abnormal beta oscillations translate into PD motor symptoms [49]. An electroencephalography study of PD patients suggested that increased cortical beta band oscillations causes abnormal synchronicity of muscle groups involved in posture holding, which increases the difficulty of initiating movement [50].

Exercise has been shown to positively impact neural oscillatory activity in PD patients and animal models as well as healthy subjects. Shi et al. [44] used a 6-hydroxydopamine (6-OHDA) rat model of PD to record local field potentials before and after exercise. They recorded lower beta band power in rats that underwent treadmill exercise, and the abnormal synchronicity within the CBGTC loop was disrupted. The exercised rats also showed functional improvements, which suggested an association between oscillatory activity regulation and motor symptoms. Results from Bougou et al. [51] support this finding and indicated that cycling can reduce beta oscillatory activity in the subthalamic nucleus of PD human patients. The authors proposed cycling as a more accessible mode of exercise for PD patients compared to walking, so it may, therefore, be a useful therapeutic modality for patients in more advanced stages of PD. In addition, although it is still uncertain how other bands of oscillation frequency are altered in PD, exercise can modulate alpha oscillatory activity in healthy young adults [52]. This change is associated with improved attention and, therefore, may contribute to alleviation of non-motor symptoms such as cognitive impairment and excessive daytime somnolence.

## 5. Cerebral Perfusion

Research into cerebral perfusion and microvasculature as they pertain to PD pathogenesis is relatively new, but it is ever-expanding, and the positive effects of exercise appear promising. Cerebral perfusion abnormalities arise early in the disease course, and this correlates with executive dysfunction [53]. Recent studies using single-photon emission computed tomography (SPECT), diffusion tensor imaging (DTI) and arterial spin labelling (ASL) have concluded that perfusion of the cortex is decreased in PD [54,55]. Conversely, there is conflicting evidence in the literature regarding perfusion of subcortical structures including the basal ganglia; while some ASL studies demonstrate perfusion is decreased, others found no change or increased perfusion [55,56,57]. At the microscopic level, loss of dopaminergic neurons results in decreased blood flow in the surrounding area [58]. It is suggested that dopamine is a regulator of blood flow to surrounding structures [55]. In particular, the SNc is highly vascularized, but post-mortem studies have shown significant loss of surrounding blood vessels [59]. These changes in perfusion can elicit further pathological sequelae such as neuroinflammation, metabolic dysfunction and increased iron deposition, manifesting in motor and non-motor PD symptoms [60].

Although there are few studies that have directly linked exercise with remediation of cerebral perfusion abnormalities in PD, it is understood that exercise can modulate cerebral blood flow, so it is likely that this is another of the mechanisms by which exercise can improve PD symptoms [31,61,62]. The mechanism behind exercise increasing cerebral blood flow is complex and multifactorial. It occurs in part due to metabolic demands in the relevant brain regions involved in the activity—as such, studies have demonstrated a positive correlation between exercise intensity and cerebral blood flow [63]—but other physiological factors such as sympathetic tone and baroreflex control also contribute [64]. Moreover, exercise may increase expression of angiogenic factors such as vascular endothelial growth factor (VEGF) for the formation of new blood vessels [65]. In other body regions, aerobic exercise has a more prominent effect than resistance exercise [66], but this comparison requires more study in PD.

## 6. Glymphatic System

The brain has very high metabolic activity with no lymphatic system, and until just over a decade ago, it was unclear how metabolic waste was removed [67]. The glymphatic system describes this missing link in the system of interstitial fluid movement and solute clearance from brain parenchyma. It is a three-stage process of firstly, CSF production and periarterial influx into the brain parenchyma; then, CSF exchange with interstitial fluid; and finally, perivenous efflux. The second stage of this process—the mixing of CSF with interstitial fluid—is particularly relevant in neurodegenerative diseases such as PD. Astrocytes are the most abundant cell type in the brain, and they play a key role in regulating CSF flow into the interstitial space and the clearance of extracellular solutes [68]. This is accomplished through expression of the aquaporin 4 (AQP4) protein on astrocytic end feet, which surround the blood vessels [69,70]. A study of DTI along the perivascular space found decreased glymphatic function in PD patients compared to healthy controls [71], and several other studies have associated decreased AQP4 expression and polarization on astrocytes with other neurodegenerative diseases such as Alzheimer’s disease [69,72]. Zhang et al. [73] determined a bidirectional relationship between AQP4 and α-Syn pathology. Loss of AQP4 increases α-Syn accumulation, and α-Syn overexpression decreases the expression and polarization of AQP4. This suggests that reversing AQP4 degradation could contribute to α-Syn clearance.

Due to the relatively recent recognition of glymphatic dysfunction in PD, research on the effects of exercise in the glymphatic system and PD are required. Extrapolation from other studies in an Alzheimer’s disease mouse model has reported that exercise can improve glymphatic clearance in both healthy and disease subjects [74,75]; hence, the notion that exercise could increase α-Syn clearance in PD is promising. Recent work by Li et al. [76] found that exercise could promote AQP4 polarization in aged mice with Alzheimer’s disease, but this study is yet to be replicated. Von Holstein-Rathlou et al. [75] suggested that improved cerebral perfusion from exercise may instead be the mechanism behind increased glymphatic clearance. As arterial pulsatility is thought to be a major driving force of glymphatic flow, increased heart rate during exercise may also contribute. It is also possible that exercise promotes protein clearance through improving sleep. Glymphatic system function is enhanced during sleep [77], and physical activity of any intensity is known to improve sleep duration and quality [78].

## 7. Neuroinflammation

Neuroinflammation is inextricably linked to the pathophysiology of PD, with complex genetic and environmental components that are yet to be completely understood [79]. Microglia, the key central nervous system immune cells, become abnormally activated early in the disease process and release pro-inflammatory cytokines [80]. Pro-inflammatory cytokines, such as interleukin-1 beta (IL-1β) and tumor necrosis factor alpha (TNF-α), are increased in PD compared to controls [81], and the levels of these cytokines are associated with the severity of motor and non-motor symptoms; to this end, pro-inflammatory cytokines have been suggested as a marker for early PD to predict disease prognosis [82]. Several factors may influence microglia activation. Many of the gene mutations and environmental exposures associated with PD exert their effects through neuroinflammation; for example, LRRK2 is expressed in microglia, and mutation is associated with innate immune system activation and pro-inflammatory cytokine release [83]. Another significant contributor is α-Syn oligomerization, which stimulates an inflammatory reaction in microglia as well as astrocytes, the most abundant glial cell in the brain [84,85]. α-Syn oligomers bind directly to toll-like receptor 2 (TLR2), causing a downstream signaling cascade and production of the pro-inflammatory cytokines TNF-α, IL-1β and IL-6 [86]. TLR4 is also upregulated in PD and is expressed by both astrocytes and microglia, which activate the NLRP3 inflammasome via a signaling cascade [85,87]. Activated microglia and astrocytes stimulate further signaling pathways that culminate in chronic inflammation, reactive oxygen species (ROS) production and reactive gliosis, resulting in neuronal injury and death [88,89].

Exercise can assist in regulating neuroinflammatory processes via several of the previously mentioned pathways, which in turn have been shown to improve the motor symptoms of PD [80,89]. Physical activity, even of low or moderate intensity, upregulates anti-inflammatory cytokines such as interleukin-10 (IL-10) and transforming growth factor beta (TGF-β) [24,90]. TGF-β is an important regulator of microglial activity; it is involved in shifting the trajectory of microglia from pro-inflammatory M1 activation to anti-inflammatory and neuroprotective M2 activation, thereby reducing the inflammatory response [91]. In mice treated with MPTP, exercise downregulated the TLR4 signaling pathway to suppress the NLRP3 inflammasome, and also blocked the activation of downstream signaling by TLR2 [87,92]. In addition, exercise can also downregulate pro-inflammatory cytokines and markers of reactive gliosis. Li et al. [93] examined levels of IL-1β in early-stage PD patients and healthy controls after Tai Chi training and brisk walking, and found that Tai Chi significantly reduced IL-1β levels. Real et al. [89] demonstrated a reduction in microglia and astrocyte activation after treadmill walking in a 6-OHDA mouse model, but this result has yet to be confirmed in human studies. Each of these mechanisms contributes to the ability of exercise to reduce the immune response and protect vulnerable dopaminergic neurons.

## 8. Gut Microbiome

Over the last few decades, research into PD etiology has expanded beyond the central nervous system. Disturbance of the gut microbiome, known as dysbiosis, has been presented as a theory. Justification for this comes from the frequency of gut-related non-motor PD symptoms such as constipation [94], and the discovery of α-Syn deposits in the enteric nervous system [95]. It has been suggested that abnormality in the gut, whether this be from inflammation or dysfunctional metabolism, can stimulate the enteric nervous system to transmit various signals to the central nervous system via the vagus nerve [96]. It has been established that the composition of the gut microbiome is altered in PD, which also affects levels of short-chain fatty acid (SCFA) metabolites produced by the bacteria [97]. The three most abundant SCFAs produced in the gut are acetate, propionate and butyrate, the last of which is the most drastically reduced in PD patients [98]. A study found that SCFA administration increased neuroinflammation and caused motor deficits in a PD mouse model [99]; however, the consensus in the current literature is in favor of the opposite finding that SCFAs may facilitate neuroprotective effects through multiple mechanisms from increasing neurotrophic factors and anti-inflammatory markers to reinforcing the blood–brain barrier [100]. Interestingly, butyrate also acts as a histone deacetylase inhibitor and has been found to reduce dopaminergic neuron damage from MPP+ via epigenetic changes that reduce neuroinflammation [101]. Decreased fecal levels of all SCFAs have been associated with the clinical severity of PD [102].

In general, it appears that physical activity can increase gut microbiota diversity and improve gut health [103,104]. In healthy rodent and human studies, exercise has been shown to increase butyrate concentration, perhaps via increasing bacteria that produce butyrate such as *Roseburia* and *Ruminococcaceae* species [105,106,107]. A study using an MPTP mouse model of PD found that exercise regulated gut dysbiosis and increased production of all SCFAs; however, this has not yet been replicated [108]. Currently available evidence suggests that the gut microbiome is influenced primarily by aerobic exercise, while resistance training has little effect [109,110]. Outcomes are also improved with higher frequency, duration and intensity of exercise [106,111]. Importantly, some studies have noted that the positive effects of exercise on the gut microbiota were reversed after cessation of a regular exercise routine [112]. It should be noted that studies of gut dysbiosis and exercise can be easily confounded by other factors such as diet [113].

## 9. Mitochondrial Dysfunction and Oxidative Stress

It is becoming increasingly evident that a major contributor to dopaminergic degeneration in PD is dysfunction of mitochondria and the resultant increase in reactive oxygen species (ROS), leading to oxidative stress. Mitochondrial dysfunction is undeniably implicated in PD pathogenesis, evident in the use of mitochondria-disrupting drugs such as MPTP and rotenone for models of PD [114]. α-Syn accumulation and oligomerization has been associated with impairment of mitochondrial complex I, which produces ROS and enacts programmed cell death mechanisms [115]. Other sources of ROS in the parkinsonian brain include excess iron in the SNc, resulting in lipid peroxidation, ferroptosis and depleted stores of antioxidants, most notably glutathione (GSH) [116,117]. Moreover, aging appears to contribute to oxidative stress. Mitochondrial dysfunction leading to increased ROS production is one key mechanism for this [114], and another is the decline in nuclear factor erythroid2-related factor 2 (Nrf2) production associated with increasing age [118]. Nrf2 is important for expression of the antioxidant enzyme glutathione peroxidase (GPX4), which restores GSH from its oxidized form, glutathione disulfide (GSSG) [119]. Together, these processes culminate in oxidative stress and cell death in dopaminergic neurons.

There is a clear connection between exercise and oxidative stress: many studies have demonstrated that muscle contraction during exercise generates ROS [120]. This may seem like a contradictory tool for reversing oxidative stress; however, exercise has been found to increase ROS only to the point where it stimulates an adaptive effect, since muscle fatigue and cardiovascular strain prevent tissue damage from excessive ROS [121]. This adaptive effect may be the reason behind decreased oxidative stress and increased blood antioxidant levels after exercise as has been shown by several studies in PD patients [122,123]. Monir et al. [124] proposed that exercise may facilitate the increase in GSH by upregulating Nrf2 expression in rats with rotenone-induced PD. Other rodent studies have found that exercise can improve mitochondrial function and turnover [125,126]. There is some controversy in the literature regarding the effects of exercise modality, intensity and length. It is generally accepted that long-term exercise results in improved outcomes compared to short-term training, but results pertaining to exercise intensity have been inconsistent between studies [127,128]. It appears that both aerobic and resistance training can provide some protection against oxidative stress, perhaps via different mechanisms [122,129].

## 10. Irisin: The Molecular Mediator?

In the case of many of the mechanisms outlined in this review, it is clear from the literature that exercise evokes change in the measured outcome, but exactly how this change comes about on a molecular level remains unclear. To help explain this, some studies have suggested the involvement of a signaling molecule known as irisin [130,131]. The precursor to irisin, the membrane protein fibronectin type III domain-containing protein 5 (FNDC5), is produced as a response to exercise in various organs such as skeletal muscle, brain and heart [132]. Once cleaved from FNDC5 and released into circulation, irisin has been shown to increase expression of neurotrophic factors such as BDNF, as well as modulating signaling pathways to reduce mitochondrial dysfunction, oxidative stress, neuroinflammatory responses and apoptosis [133,134,135]. It has been suggested that irisin could be exogenously administered to mimic the neuroprotective effects of exercise since it has been shown to cross the blood–brain barrier [136]. This concept is still in the early stages of research, and while initial findings are encouraging, more research is needed [131,137].

## 11. Discussion

**Figure 1:**
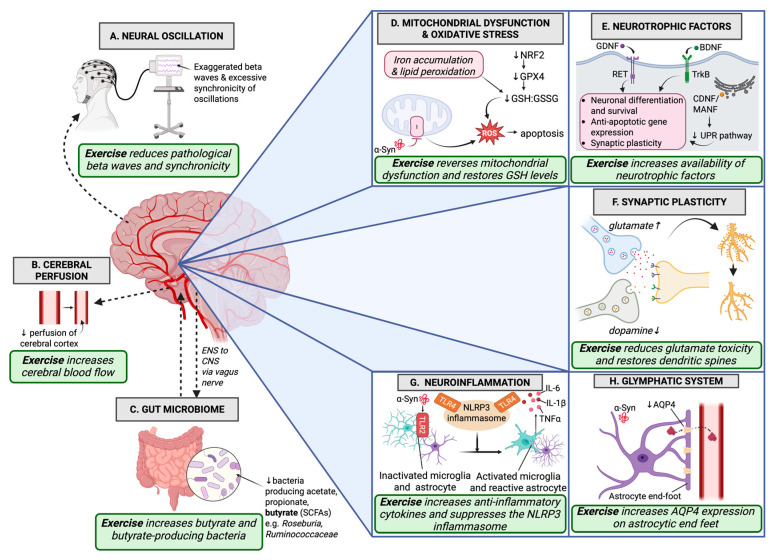
Schematic figure outlining the pathophysiological mechanisms in Parkinson’s disease (PD) that are modulated by exercise. (**A**) Pathological beta waves and synchronization of neural oscillations on electroencephalography can be reduced by exercise. (**B**) Both macroscopic and microscopic vessel perfusion is decreased in PD, and exercise can restore perfusion. (**C**) Gut dysbiosis is a proposed contributor to PD etiology, leading to decreased levels of beneficial short-chain fatty acids (SCFAs) such as butyrate; exercise can increase both butyrate and butyrate-producing bacteria. (**D**) Exercise reverses the effects of mitochondrial dysfunction and acts via nuclear factor erythroid2-related factor 2 (Nrf2) to increase glutathione peroxidase (GPX4) levels and restore the glutathione-to-glutathione disulfide ratio (GSH/GSSG), resulting in reduced oxidative damage. (**E**) Exercise can increase the levels of protective neurotrophic factors in the brain, including brain-derived neurotrophic factor (BDNF), which acts on tropomyosin-related kinase B (TrkB) receptors, glial cell line-derived neurotrophic factor (GDNF), which acts on rearranged during transfection (RET) receptors, and cerebral dopamine neurotrophic factor/mesencephalic astrocyte-derived neurotrophic factor (CDNF/MANF), which regulates the Unfolded Protein Response (UPR). (**F**) Dopaminergic cell death leads to a lack of dopamine to oppose glutamate at synapses, causing excitotoxicity manifesting as loss of dendritic spine density; exercise can reduce excitotoxicity and restore dendritic spines. (**G**) Alpha-synuclein (α-Syn) binding toll-like receptor 2 (TLR2) on microglia and upregulation of toll-like receptor 4 (TLR4) causing activation of the NOD-, LRR- and pyrin domain-containing protein 3 (NLRP3) inflammasome result in increased microglia activation and excessive pro-inflammatory cytokine release; exercise can increase anti-inflammatory cytokines to combat this, and can suppress the NLRP3 inflammasome. (**H**) Exercise increases aquaporin 4 (AQP4) receptors on astrocytic end-feet, which are downregulated in PD, and may augment glymphatic clearance by increasing arterial pulsatility, increasing clearance of proteins from the brain parenchyma.

It is undeniable that exercise has merit as a therapeutic tool for PD, with widespread impacts on many aspects of disease physiology from the dopaminergic synapse to central nervous system perfusion. These mechanisms provide a basis for the benefits of exercise in many neurodegenerative diseases, combating motor and non-motor symptoms as well as building strength in people experiencing age-related frailty and motor decline. The physiological effects and potential therapeutic mechanisms of exercise in the context of PD are summarized in Figure 1. Currently, very few studies have investigated different modalities, intensity and length of exercise in the context of PD, thus more research is required to further define optimal exercise regimes in PD therapy. Based on current studies, the consensus is that high-intensity exercise produces the most significant outcomes, and aerobic exercise is generally more effective than resistance training. However, many studies highlight that exercise of any intensity or format can still be beneficial [9,138].

One concern regarding exercise as a therapeutic option is the accessibility and suitability in individuals with advanced stages of PD. While some forms of aerobic exercise, such as running, may be more beneficial for patients in early stages of the disease [139], other modalities, including dance or virtual reality-assisted exercise, may be more accessible and safer for patients with severe symptoms [140,141]. Mind–body exercises such as yoga or Tai Chi can also be considered as lower impact alternatives [93,142]. A recent study demonstrated the beneficial effects of high-intensity exercise facilitated by immersive virtual reality technology, finding improvements in functional capacity, quality of life and disease progression [139]. It should be noted that most types of exercise incur some risk of adverse effects, primarily falls; hence, precautions such as seated exercise are required especially in those with more severe disease [138]. Novel technologies such as virtual reality will likely contribute to the mitigation of these risks [139].

A limitation in the current evidence on therapeutic mechanisms of exercise in PD is the ongoing reliance on predominant mouse or rat models of PD—typically induced with neurotoxins such as MPTP or 6-OHDA—rather than human studies. These mouse models do not fully represent the disease course and symptoms of idiopathic PD nor reflect specific physiological and pathophysiological responses to interventions such as exercise [143]. Nevertheless, each of the outlined mechanisms is supported by limited human studies that encompass the comprehensive use of potential biomarkers in the pathophysiology of disease and neurodegeneration. More human studies should be a priority for future research to support current animal data.

## 12. Conclusions

PD is a complex condition with no definitive treatment, so any therapies that may delay the disease course are valuable. There exists a vast amount of literature validating the clinical impact of exercise in PD. This review provides a basis for the far-reaching and likely beneficial effects of exercise in PD that range from gut dysbiosis to neuroinflammation, and further justifies the need for exercise to form a cornerstone in PD therapeutics. Future research should supplement presently available animal-based studies. More human-based research studies will be invaluable in further elucidating the most appropriate form and intensity of exercise in PD, and this may vary based on the various stages of the disease.

## Data Availability

No new data were created or analyzed in this study.

## References

[1] Bloem B.R., Okun M.S., Klein C. (2021). Parkinson’s disease. Lancet.

[2] Wang J., Wang F., Mai D., Qu S. (2020). Molecular Mechanisms of Glutamate Toxicity in Parkinson’s Disease. Front. Neurosci..

[3] Dong-Chen X., Yong C., Yang X., Chen-Yu S., Li-Hua P. (2023). Signaling pathways in Parkinson’s disease: Molecular mechanisms and therapeutic interventions. Signal Transduct. Target. Ther..

[4] Farrow S.L., Cooper A.A., O’Sullivan J.M. (2022). Redefining the hypotheses driving Parkinson’s diseases research. npj Parkinson’s Dis..

[5] Bennett D.A., Beckett L.A., Murray A.M., Shannon K.M., Goetz C.G., Pilgrim D.M., Evans D.A. (1996). Prevalence of parkinsonian signs and associated mortality in a community population of older people. N. Engl. J. Med..

[6] Dorsey E.R., De Miranda B.R., Horsager J., Borghammer P. (2024). The Body, the Brain, the Environment, and Parkinson’s Disease. J. Parkinson’s Dis..

[7] Zapanta K., Schroeder E.T., Fisher B.E. (2022). Rethinking Parkinson Disease: Exploring Gut-Brain Interactions and the Potential Role of Exercise. Phys. Ther..

[8] Feng Y.S., Yang S.D., Tan Z.X., Wang M.M., Xing Y., Dong F., Zhang F. (2020). The benefits and mechanisms of exercise training for Parkinson’s disease. Life Sci..

[9] Bispo D., Lins C., Hawkes K.L., Tripp S., Khoo T.K. (2024). The Positive Effects of Physical Activity on Quality of Life in Parkinson’s Disease: A Systematic Review. Geriatrics.

[10] Amara A.W., Memon A.A. (2018). Effects of Exercise on Non-motor Symptoms in Parkinson’s Disease. Clin. Ther..

[11] Dauwan M., Begemann M.J.H., Slot M.I.E., Lee E.H.M., Scheltens P., Sommer I.E.C. (2021). Physical exercise improves quality of life, depressive symptoms, and cognition across chronic brain disorders: A transdiagnostic systematic review and meta-analysis of randomized controlled trials. J. Neurol..

[12] Sujkowski A., Hong L., Wessells R.J., Todi S.V. (2022). The protective role of exercise against age-related neurodegeneration. Ageing Res. Rev..

[13] Palasz E., Wysocka A., Gasiorowska A., Chalimoniuk M., Niewiadomski W., Niewiadomska G. (2020). BDNF as a Promising Therapeutic Agent in Parkinson’s Disease. Int. J. Mol. Sci..

[14] Albert K., Raymundo D.P., Panhelainen A., Eesmaa A., Shvachiy L., Araujo G.R., Chmielarz P., Yan X., Singh A., Cordeiro Y. (2021). Cerebral dopamine neurotrophic factor reduces alpha-synuclein aggregation and propagation and alleviates behavioral alterations in vivo. Mol. Ther..

[15] Lohelaid H., Saarma M., Airavaara M. (2024). CDNF and ER stress: Pharmacology and therapeutic possibilities. Pharmacol. Ther..

[16] Houlton J., Abumaria N., Hinkley S.F.R., Clarkson A.N. (2019). Therapeutic Potential of Neurotrophins for Repair After Brain Injury: A Helping Hand From Biomaterials. Front. Neurosci..

[17] Palasz E., Niewiadomski W., Gasiorowska A., Wysocka A., Stepniewska A., Niewiadomska G. (2019). Exercise-Induced Neuroprotection and Recovery of Motor Function in Animal Models of Parkinson’s Disease. Front. Neurol..

[18] Ateaque S., Merkouris S., Barde Y.A. (2023). Neurotrophin signalling in the human nervous system. Front. Mol. Neurosci..

[19] Kaplan D.R., Miller F.D. (2000). Neurotrophin signal transduction in the nervous system. Curr. Opin. Neurobiol..

[20] Reichardt L.F. (2006). Neurotrophin-regulated signalling pathways. Philos. Trans. R. Soc. Lond. B Biol. Sci..

[21] Bonanni R., Cariati I., Tarantino U., D’Arcangelo G., Tancredi V. (2022). Physical Exercise and Health: A Focus on Its Protective Role in Neurodegenerative Diseases. J. Funct. Morphol. Kinesiol..

[22] Barker R.A., Bjorklund A., Gash D.M., Whone A., Van Laar A., Kordower J.H., Bankiewicz K., Kieburtz K., Saarma M., Booms S. (2020). GDNF and Parkinson’s Disease: Where Next? A Summary from a Recent Workshop. J. Parkinson’s Dis..

[23] Pakarinen E., Lindholm P. (2023). CDNF and MANF in the brain dopamine system and their potential as treatment for Parkinson’s disease. Front. Psychiatry.

[24] Leem Y.H., Park J.S., Park J.E., Kim D.Y., Kim H.S. (2023). Suppression of neuroinflammation and alpha-synuclein oligomerization by rotarod walking exercise in subacute MPTP model of Parkinson’s disease. Neurochem. Int..

[25] Da Silva W.A.B., Ferreira Oliveira K., Caroline Vitorino L., Ferreira Romao L., Allodi S., Lourenco Correa C. (2021). Physical exercise increases the production of tyrosine hydroxylase and CDNF in the spinal cord of a Parkinson’s disease mouse model. Neurosci. Lett..

[26] Palasz E., Niewiadomski W., Gasiorowska A., Mietelska-Porowska A., Niewiadomska G. (2019). Neuroplasticity and Neuroprotective Effect of Treadmill Training in the Chronic Mouse Model of Parkinson’s Disease. Neural Plast..

[27] Fontanesi C., Kvint S., Frazzitta G., Bera R., Ferrazzoli D., Di Rocco A., Rebholz H., Friedman E., Pezzoli G., Quartarone A. (2016). Intensive Rehabilitation Enhances Lymphocyte BDNF-TrkB Signaling in Patients with Parkinson’s Disease. Neurorehabil. Neural Repair..

[28] Paterno A., Polsinelli G., Federico B. (2024). Changes of brain-derived neurotrophic factor (BDNF) levels after different exercise protocols: A systematic review of clinical studies in Parkinson’s disease. Front. Physiol..

[29] Fallah Mohammadi Z., Falah Mohammadi H., Patel D.I. (2019). Comparing the effects of progressive and mild intensity treadmill running protocols on neuroprotection of parkinsonian rats. Life Sci..

[30] McCullough M.J., Gyorkos A.M., Spitsbergen J.M. (2013). Short-term exercise increases GDNF protein levels in the spinal cord of young and old rats. Neuroscience.

[31] Petzinger G.M., Holschneider D.P., Fisher B.E., McEwen S., Kintz N., Halliday M., Toy W., Walsh J.W., Beeler J., Jakowec M.W. (2015). The Effects of Exercise on Dopamine Neurotransmission in Parkinson’s Disease: Targeting Neuroplasticity to Modulate Basal Ganglia Circuitry. Brain Plast..

[32] Albin R.L., Young A.B., Penney J.B. (1989). The functional anatomy of basal ganglia disorders. Trends Neurosci..

[33] Zheng X., Huang Z., Zhu Y., Liu B., Chen Z., Chen T., Jia L., Li Y., Lei W. (2019). Increase in Glutamatergic Terminals in the Striatum Following Dopamine Depletion in a Rat Model of Parkinson’s Disease. Neurochem. Res..

[34] Deutch A.Y., Colbran R.J., Winder D.J. (2007). Striatal plasticity and medium spiny neuron dendritic remodeling in parkinsonism. Parkinsonism Relat. Disord..

[35] Segal M., Andersen P. (2000). Dendritic spines shaped by synaptic activity. Curr. Opin. Neurobiol..

[36] Shin M.S., Jeong H.Y., An D.I., Lee H.Y., Sung Y.H. (2016). Treadmill exercise facilitates synaptic plasticity on dopaminergic neurons and fibers in the mouse model with Parkinson’s disease. Neurosci. Lett..

[37] Feng Y., Zhou S., Sun J. (2021). Exercise increases striatal Glu reuptake and improves motor dysfunction in 6-OHDA-induced Parkinson’s disease rats. Exp. Brain Res..

[38] Alarcon T.A., Presti-Silva S.M., Simoes A.P.T., Ribeiro F.M., Pires R.G.W. (2023). Molecular mechanisms underlying the neuroprotection of environmental enrichment in Parkinson’s disease. Neural Regen. Res..

[39] De Laat B., Hoye J., Stanley G., Hespeler M., Ligi J., Mohan V., Wooten D.W., Zhang X., Nguyen T.D., Key J. (2024). Intense exercise increases dopamine transporter and neuromelanin concentrations in the substantia nigra in Parkinson’s disease. npj Parkinson’s Dis..

[40] Kasanga E.A., Soto I., Centner A., McManus R., Shifflet M.K., Navarrete W., Han Y., Lisk J., Ehrhardt T., Wheeler K. (2024). Moderate intensity aerobic exercise alleviates motor deficits in 6-OHDA lesioned rats and reduces serum levels of biomarkers of Parkinson’s disease severity without recovery of striatal dopamine or tyrosine hydroxylase. Exp. Neurol..

[41] Kintz N., Petzinger G.M., Jakowec M.W. (2017). Treadmill exercise modifies dopamine receptor expression in the prefrontal cortex of the 1-methyl-4-phenyl-1,2,3,6-tetrahydropyridine-lesioned mouse model of Parkinson’s disease. Neuroreport.

[42] VanLeeuwen J.E., Petzinger G.M., Walsh J.P., Akopian G.K., Vuckovic M., Jakowec M.W. (2010). Altered AMPA receptor expression with treadmill exercise in the 1-methyl-4-phenyl-1,2,3,6-tetrahydropyridine-lesioned mouse model of basal ganglia injury. J. Neurosci. Res..

[43] Gergin S., Kirazli O., Boraci H., Yildiz S.D., Yananli H.R., Sehirli U.S. (2023). The effects of regular swimming exercise and melatonin on the neurons localized in the striatum of hemiparkinsonian rats. Anat. Sci. Int..

[44] Shi K., Liu X., Hou L., Qiao D., Peng Y. (2021). Exercise Improves Movement by Regulating the Plasticity of Cortical Function in Hemiparkinsonian Rats. Front. Aging Neurosci..

[45] Asadi A., Madadi Asl M., Vahabie A.H., Valizadeh A. (2022). The Origin of Abnormal Beta Oscillations in the Parkinsonian Corticobasal Ganglia Circuits. Parkinson’s Dis..

[46] Galvan A., Devergnas A., Wichmann T. (2015). Alterations in neuronal activity in basal ganglia-thalamocortical circuits in the parkinsonian state. Front. Neuroanat..

[47] Costa R.M., Lin S.C., Sotnikova T.D., Cyr M., Gainetdinov R.R., Caron M.G., Nicolelis M.A. (2006). Rapid alterations in corticostriatal ensemble coordination during acute dopamine-dependent motor dysfunction. Neuron.

[48] Mallet N., Pogosyan A., Sharott A., Csicsvari J., Bolam J.P., Brown P., Magill P.J. (2008). Disrupted dopamine transmission and the emergence of exaggerated beta oscillations in subthalamic nucleus and cerebral cortex. J. Neurosci..

[49] Halje P., Brys I., Mariman J.J., da Cunha C., Fuentes R., Petersson P. (2019). Oscillations in cortico-basal ganglia circuits: Implications for Parkinson’s disease and other neurologic and psychiatric conditions. J. Neurophysiol..

[50] Simpson T.G., Godfrey W., Torrecillos F., He S., Herz D.M., Oswal A., Muthuraman M., Pogosyan A., Tan H. (2024). Cortical beta oscillations help synchronise muscles during static posture holding in healthy motor control. Neuroimage.

[51] Bougou V., Vanhoyland M., Decramer T., Van Hoylandt A., Smeijers S., Nuttin B., De Vloo P., Vandenberghe W., Nieuwboer A., Janssen P. (2024). Active and Passive Cycling Decrease Subthalamic beta Oscillations in Parkinson’s Disease. Mov. Disord..

[52] Chaire A., Becke A., Duzel E. (2020). Effects of Physical Exercise on Working Memory and Attention-Related Neural Oscillations. Front. Neurosci..

[53] Firbank M.J., Molloy S., McKeith I.G., Burn D.J., O’Brien J.T. (2005). Longitudinal change in 99mTcHMPAO cerebral perfusion SPECT in Parkinson’s disease over one year. J. Neurol. Neurosurg. Psychiatry.

[54] Rane S., Koh N., Oakley J., Caso C., Zabetian C.P., Cholerton B., Montine T.J., Grabowski T. (2020). Arterial spin labeling detects perfusion patterns related to motor symptoms in Parkinson’s disease. Parkinsonism Relat. Disord..

[55] Liu Z., Zhang Y., Wang H., Xu D., You H., Zuo Z., Feng F. (2022). Altered cerebral perfusion and microstructure in advanced Parkinson’s disease and their associations with clinical features. Neurol. Res..

[56] Pelizzari L., Lagana M.M., Di Tella S., Rossetto F., Bergsland N., Nemni R., Clerici M., Baglio F. (2019). Combined Assessment of Diffusion Parameters and Cerebral Blood Flow Within Basal Ganglia in Early Parkinson’s Disease. Front. Aging Neurosci..

[57] Erro R., Ponticorvo S., Manara R., Barone P., Picillo M., Scannapieco S., Cicarelli G., Squillante M., Volpe G., Esposito F. (2020). Subcortical atrophy and perfusion patterns in Parkinson disease and multiple system atrophy. Parkinsonism Relat. Disord..

[58] Liu J., Min L., Liu R., Zhang X., Wu M., Di Q., Ma X. (2023). The effect of exercise on cerebral blood flow and executive function among young adults: A double-blinded randomized controlled trial. Sci. Rep..

[59] Issidorides M.R. (1971). Neuronal vascular relationships in the zona compacta of normal and parkinsonian substantia nigra. Brain Res..

[60] Zhang C., Wu B., Wang X., Chen C., Zhao R., Lu H., Zhu H., Xue B., Liang H., Sethi S.K. (2020). Vascular, flow and perfusion abnormalities in Parkinson’s disease. Parkinsonism Relat. Disord..

[61] Smith J.C., Paulson E.S., Cook D.B., Verber M.D., Tian Q. (2010). Detecting changes in human cerebral blood flow after acute exercise using arterial spin labeling: Implications for fMRI. J. Neurosci. Methods.

[62] Secher N.H., Seifert T., Van Lieshout J.J. (2008). Cerebral blood flow and metabolism during exercise: Implications for fatigue. J. Appl. Physiol..

[63] Mekari S., Neyedli H.F., Fraser S., O’Brien M.W., Martins R., Evans K., Earle M., Aucoin R., Chiekwe J., Hollohan Q. (2020). High-Intensity Interval Training Improves Cognitive Flexibility in Older Adults. Brain Sci..

[64] Ogoh S., Ainslie P.N. (2009). Regulatory mechanisms of cerebral blood flow during exercise: New concepts. Exerc. Sport Sci. Rev..

[65] Viboolvorakul S., Patumraj S. (2014). Exercise training could improve age-related changes in cerebral blood flow and capillary vascularity through the upregulation of VEGF and eNOS. BioMed Res. Int..

[66] Kwak S.E., Lee J.H., Zhang D., Song W. (2018). Angiogenesis: Focusing on the effects of exercise in aging and cancer. J. Exerc. Nutr. Biochem..

[67] Iliff J.J., Wang M., Liao Y., Plogg B.A., Peng W., Gundersen G.A., Benveniste H., Vates G.E., Deane R., Goldman S.A. (2012). A paravascular pathway facilitates CSF flow through the brain parenchyma and the clearance of interstitial solutes, including amyloid beta. Sci. Transl. Med..

[68] Plog B.A., Nedergaard M. (2018). The Glymphatic System in Central Nervous System Health and Disease: Past, Present, and Future. Annu. Rev. Pathol..

[69] Szlufik S., Kopec K., Szleszkowski S., Koziorowski D. (2024). Glymphatic System Pathology and Neuroinflammation as Two Risk Factors of Neurodegeneration. Cells.

[70] Mestre H., Hablitz L.M., Xavier A.L., Feng W., Zou W., Pu T., Monai H., Murlidharan G., Castellanos Rivera R.M., Simon M.J. (2018). Aquaporin-4-dependent glymphatic solute transport in the rodent brain. eLife.

[71] Wood K.H., Nenert R., Miften A.M., Kent G.W., Sleyster M., Memon R.A., Joop A., Pilkington J., Memon A.A., Wilson R.N. (2024). Diffusion Tensor Imaging-Along the Perivascular-Space Index Is Associated with Disease Progression in Parkinson’s Disease. Mov. Disord..

[72] Zeppenfeld D.M., Simon M., Haswell J.D., D’Abreo D., Murchison C., Quinn J.F., Grafe M.R., Woltjer R.L., Kaye J., Iliff J.J. (2017). Association of Perivascular Localization of Aquaporin-4 with Cognition and Alzheimer Disease in Aging Brains. JAMA Neurol..

[73] Zhang Y., Zhang C., He X.Z., Li Z.H., Meng J.C., Mao R.T., Li X., Xue R., Gui Q., Zhang G.X. (2023). Interaction Between the Glymphatic System and alpha-Synuclein in Parkinson’s Disease. Mol. Neurobiol..

[74] He X.F., Liu D.X., Zhang Q., Liang F.Y., Dai G.Y., Zeng J.S., Pei Z., Xu G.Q., Lan Y. (2017). Voluntary Exercise Promotes Glymphatic Clearance of Amyloid Beta and Reduces the Activation of Astrocytes and Microglia in Aged Mice. Front. Mol. Neurosci..

[75] Von Holstein-Rathlou S., Petersen N.C., Nedergaard M. (2018). Voluntary running enhances glymphatic influx in awake behaving, young mice. Neurosci. Lett..

[76] Li M., Xu J., Li L., Zhang L., Zuo Z., Feng Y., He X., Hu X. (2024). Voluntary wheel exercise improves glymphatic clearance and ameliorates colitis-associated cognitive impairment in aged mice by inhibiting TRPV4-induced astrocytic calcium activity. Exp. Neurol..

[77] Jessen N.A., Munk A.S., Lundgaard I., Nedergaard M. (2015). The Glymphatic System: A Beginner’s Guide. Neurochem. Res..

[78] Dolezal B.A., Neufeld E.V., Boland D.M., Martin J.L., Cooper C.B. (2017). Interrelationship between Sleep and Exercise: A Systematic Review. Adv. Prev. Med..

[79] Tansey M.G., Wallings R.L., Houser M.C., Herrick M.K., Keating C.E., Joers V. (2022). Inflammation and immune dysfunction in Parkinson disease. Nat. Rev. Immunol..

[80] Mee-Inta O., Zhao Z.W., Kuo Y.M. (2019). Physical Exercise Inhibits Inflammation and Microglial Activation. Cells.

[81] Qu Y., Li J., Qin Q., Wang D., Zhao J., An K., Mao Z., Min Z., Xiong Y., Li J. (2023). A systematic review and meta-analysis of inflammatory biomarkers in Parkinson’s disease. npj Parkinson’s Dis..

[82] Williams-Gray C.H., Wijeyekoon R., Yarnall A.J., Lawson R.A., Breen D.P., Evans J.R., Cummins G.A., Duncan G.W., Khoo T.K., Burn D.J. (2016). Serum immune markers and disease progression in an incident Parkinson’s disease cohort (ICICLE-PD). Mov. Disord..

[83] Gillardon F., Schmid R., Draheim H. (2012). Parkinson’s disease-linked leucine-rich repeat kinase 2(R1441G) mutation increases proinflammatory cytokine release from activated primary microglial cells and resultant neurotoxicity. Neuroscience.

[84] Wang S., Chu C.H., Stewart T., Ginghina C., Wang Y., Nie H., Guo M., Wilson B., Hong J.S., Zhang J. (2015). alpha-Synuclein, a chemoattractant, directs microglial migration via H2O2-dependent Lyn phosphorylation. Proc. Natl. Acad. Sci. USA.

[85] Fellner L., Irschick R., Schanda K., Reindl M., Klimaschewski L., Poewe W., Wenning G.K., Stefanova N. (2013). Toll-like receptor 4 is required for alpha-synuclein dependent activation of microglia and astroglia. Glia.

[86] Kim C., Ho D.H., Suk J.E., You S., Michael S., Kang J., Joong Lee S., Masliah E., Hwang D., Lee H.J. (2013). Neuron-released oligomeric alpha-synuclein is an endogenous agonist of TLR2 for paracrine activation of microglia. Nat. Commun..

[87] Wang W., Lv Z., Gao J., Liu M., Wang Y., Tang C., Xiang J. (2021). Treadmill exercise alleviates neuronal damage by suppressing NLRP3 inflammasome and microglial activation in the MPTP mouse model of Parkinson’s disease. Brain Res. Bull..

[88] Block M.L., Zecca L., Hong J.S. (2007). Microglia-mediated neurotoxicity: Uncovering the molecular mechanisms. Nat. Rev. Neurosci..

[89] Real C.C., Garcia P.C., Britto L.R.G. (2017). Treadmill Exercise Prevents Increase of Neuroinflammation Markers Involved in the Dopaminergic Damage of the 6-OHDA Parkinson’s Disease Model. J. Mol. Neurosci..

[90] Szymura J., Kubica J., Wiecek M., Pera J. (2020). The Immunomodulary Effects of Systematic Exercise in Older Adults and People with Parkinson’s Disease. J. Clin. Med..

[91] Zhou X., Spittau B., Krieglstein K. (2012). TGFbeta signalling plays an important role in IL4-induced alternative activation of microglia. J. Neuroinflamm..

[92] Jang Y., Koo J.H., Kwon I., Kang E.B., Um H.S., Soya H., Lee Y., Cho J.Y. (2017). Neuroprotective effects of endurance exercise against neuroinflammation in MPTP-induced Parkinson’s disease mice. Brain Res..

[93] Li G., Huang P., Cui S.S., Tan Y.Y., He Y.C., Shen X., Jiang Q.Y., Huang P., He G.Y., Li B.Y. (2022). Mechanisms of motor symptom improvement by long-term Tai Chi training in Parkinson’s disease patients. Transl. Neurodegener..

[94] Postuma R.B., Gagnon J.F., Pelletier A., Montplaisir J. (2013). Prodromal autonomic symptoms and signs in Parkinson’s disease and dementia with Lewy bodies. Mov. Disord..

[95] Beach T.G., Adler C.H., Sue L.I., Vedders L., Lue L., White Iii C.L., Akiyama H., Caviness J.N., Shill H.A., Sabbagh M.N. (2010). Multi-organ distribution of phosphorylated alpha-synuclein histopathology in subjects with Lewy body disorders. Acta Neuropathol..

[96] Goehler L.E., Busch C.R., Tartaglia N., Relton J., Sisk D., Maier S.F., Watkins L.R. (1995). Blockade of cytokine induced conditioned taste aversion by subdiaphragmatic vagotomy: Further evidence for vagal mediation of immune-brain communication. Neurosci. Lett..

[97] Aho V.T.E., Houser M.C., Pereira P.A.B., Chang J., Rudi K., Paulin L., Hertzberg V., Auvinen P., Tansey M.G., Scheperjans F. (2021). Relationships of gut microbiota, short-chain fatty acids, inflammation, and the gut barrier in Parkinson’s disease. Mol. Neurodegener..

[98] Cirstea M.S., Yu A.C., Golz E., Sundvick K., Kliger D., Radisavljevic N., Foulger L.H., Mackenzie M., Huan T., Finlay B.B. (2020). Microbiota Composition and Metabolism Are Associated with Gut Function in Parkinson’s Disease. Mov. Disord..

[99] Sampson T.R., Debelius J.W., Thron T., Janssen S., Shastri G.G., Ilhan Z.E., Challis C., Schretter C.E., Rocha S., Gradinaru V. (2016). Gut Microbiota Regulate Motor Deficits and Neuroinflammation in a Model of Parkinson’s Disease. Cell.

[100] Silva Y.P., Bernardi A., Frozza R.L. (2020). The Role of Short-Chain Fatty Acids From Gut Microbiota in Gut-Brain Communication. Front. Endocrinol..

[101] Kidd S.K., Schneider J.S. (2010). Protection of dopaminergic cells from MPP^+^-mediated toxicity by histone deacetylase inhibition. Brain Res..

[102] Chen S.J., Chen C.C., Liao H.Y., Lin Y.T., Wu Y.W., Liou J.M., Wu M.S., Kuo C.H., Lin C.H. (2022). Association of Fecal and Plasma Levels of Short-Chain Fatty Acids with Gut Microbiota and Clinical Severity in Patients with Parkinson Disease. Neurology.

[103] Yang W., Liu Y., Yang G., Meng B., Yi Z., Yang G., Chen M., Hou P., Wang H., Xu X. (2021). Moderate-Intensity Physical Exercise Affects the Exercise Performance and Gut Microbiota of Mice. Front. Cell Infect. Microbiol..

[104] Munukka E., Ahtiainen J.P., Puigbo P., Jalkanen S., Pahkala K., Keskitalo A., Kujala U.M., Pietila S., Hollmen M., Elo L. (2018). Six-Week Endurance Exercise Alters Gut Metagenome That Is not Reflected in Systemic Metabolism in Over-weight Women. Front. Microbiol..

[105] Mitchell C.M., Davy B.M., Hulver M.W., Neilson A.P., Bennett B.J., Davy K.P. (2019). Does Exercise Alter Gut Microbial Composition? A Systematic Review. Med. Sci. Sports Exerc..

[106] Batacan R.B., Fenning A.S., Dalbo V.J., Scanlan A.T., Duncan M.J., Moore R.J., Stanley D. (2017). A gut reaction: The combined influence of exercise and diet on gastrointestinal microbiota in rats. J. Appl. Microbiol..

[107] Matsumoto M., Inoue R., Tsukahara T., Ushida K., Chiji H., Matsubara N., Hara H. (2008). Voluntary running exercise alters microbiota composition and increases n-butyrate concentration in the rat cecum. Biosci. Biotechnol. Biochem..

[108] Wang Y., Pu Z., Zhang Y., Du Z., Guo Z., Bai Q. (2023). Exercise training has a protective effect in 1-methyl-4-phenyl-1,2,3,6-tetrahydropyridine mice model with improved neural and intestinal pathology and modified intestinal flora. Behav. Brain Res..

[109] Bycura D., Santos A.C., Shiffer A., Kyman S., Winfree K., Sutliffe J., Pearson T., Sonderegger D., Cope E., Caporaso J.G. (2021). Impact of Different Exercise Modalities on the Human Gut Microbiome. Sports.

[110] Moore J.H., Smith K.S., Chen D., Lamb D.A., Smith M.A., Osburn S.C., Ruple B.A., Morrow C.D., Huggins K.W., McDonald J.R. (2022). Exploring the Effects of Six Weeks of Resistance Training on the Fecal Microbiome of Older Adult Males: Secondary Analysis of a Peanut Protein Supplemented Randomized Controlled Trial. Sports.

[111] Boytar A.N., Skinner T.L., Wallen R.E., Jenkins D.G., Dekker Nitert M. (2023). The Effect of Exercise Prescription on the Human Gut Microbiota and Comparison between Clinical and Apparently Healthy Populations: A Systematic Review. Nutrients.

[112] Allen J.M., Mailing L.J., Niemiro G.M., Moore R., Cook M.D., White B.A., Holscher H.D., Woods J.A. (2018). Exercise Alters Gut Microbiota Composition and Function in Lean and Obese Humans. Med. Sci. Sports Exerc..

[113] Zhang P. (2022). Influence of Foods and Nutrition on the Gut Microbiome and Implications for Intestinal Health. Int. J. Mol. Sci..

[114] Henrich M.T., Oertel W.H., Surmeier D.J., Geibl F.F. (2023). Mitochondrial dysfunction in Parkinson’s disease—A key disease hallmark with therapeutic potential. Mol. Neurodegener..

[115] Mohan S., Alhazmi H.A., Hassani R., Khuwaja G., Maheshkumar V.P., Aldahish A., Chidambaram K. (2024). Role of ferroptosis pathways in neuroinflammation and neurological disorders: From pathogenesis to treatment. Heliyon.

[116] Dias V., Junn E., Mouradian M.M. (2013). The role of oxidative stress in Parkinson’s disease. J. Parkinson’s Dis..

[117] Niu C., Dong M., Niu Y. (2024). Role of Glutathione in Parkinson’s Disease Pathophysiology and Therapeutic Potential of Polyphenols. Phytother. Res..

[118] Johnson D.A., Johnson J.A. (2015). Nrf2—A therapeutic target for the treatment of neurodegenerative diseases. Free Radic. Biol. Med..

[119] Suh J.H., Shenvi S.V., Dixon B.M., Liu H., Jaiswal A.K., Liu R.M., Hagen T.M. (2004). Decline in transcriptional activity of Nrf2 causes age-related loss of glutathione synthesis, which is reversible with lipoic acid. Proc. Natl. Acad. Sci. USA.

[120] Powers S.K., Deminice R., Ozdemir M., Yoshihara T., Bomkamp M.P., Hyatt H. (2020). Exercise-induced oxidative stress: Friend or foe?. J. Sport. Health Sci..

[121] Ji L.L., Kang C., Zhang Y. (2016). Exercise-induced hormesis and skeletal muscle health. Free Radic. Biol. Med..

[122] Tsai C.L., Chien C.Y., Pan C.Y., Tseng Y.T., Wang T.C., Lin T.K. (2025). Effects of long-term Tai Chi vs. aerobic exercise on antioxidant activity and cognitive function in individuals with Parkinson’s disease. Behav. Brain Res..

[123] Bloomer R.J., Schilling B.K., Karlage R.E., Ledoux M.S., Pfeiffer R.F., Callegari J. (2008). Effect of resistance training on blood oxidative stress in Parkinson disease. Med. Sci. Sports Exerc..

[124] Monir D.M., Mahmoud M.E., Ahmed O.G., Rehan I.F., Abdelrahman A. (2020). Forced exercise activates the NrF2 pathway in the striatum and ameliorates motor and behavioral manifestations of Parkinson’s disease in rotenone-treated rats. Behav. Brain Funct..

[125] Koo J.H., Cho J.Y., Lee U.B. (2017). Treadmill exercise alleviates motor deficits and improves mitochondrial import machinery in an MPTP-induced mouse model of Parkinson’s disease. Exp. Gerontol..

[126] Chuang C.S., Chang J.C., Cheng F.C., Liu K.H., Su H.L., Liu C.S. (2017). Modulation of mitochondrial dynamics by treadmill training to improve gait and mitochondrial deficiency in a rat model of Parkinson’s disease. Life Sci..

[127] Tutakhail A., Nazary Q.A., Lebsir D., Kerdine-Romer S., Coudore F. (2018). Induction of brain Nrf2-HO-1 pathway and antinociception after different physical training paradigms in mice. Life Sci..

[128] Tung Y.T., Liao Y.C., Yeh T.H., Tsao S.P., Chang C.C., Shih W.T., Huang H.Y. (2024). 10 weeks low intensity treadmill exercise intervention ameliorates motor deficits and sustains muscle mass via decreasing oxidative damage and increasing mitochondria function in a rat model of Parkinson’s disease. Life Sci..

[129] Pinho R.A., Aguiar A.S., Radak Z. (2019). Effects of Resistance Exercise on Cerebral Redox Regulation and Cognition: An Interplay Between Muscle and Brain. Antioxidants.

[130] Chow L.S., Gerszten R.E., Taylor J.M., Pedersen B.K., van Praag H., Trappe S., Febbraio M.A., Galis Z.S., Gao Y., Haus J.M. (2022). Exerkines in health, resilience and disease. Nat. Rev. Endocrinol..

[131] Avgerinos K.I., Liu J., Dalamaga M. (2023). Could exercise hormone irisin be a therapeutic agent against Parkinson’s and other neurodegenerative diseases?. Metabol. Open.

[132] Ferrer-Martinez A., Ruiz-Lozano P., Chien K.R. (2002). Mouse PeP: A novel peroxisomal protein linked to myoblast differentiation and development. Dev. Dyn..

[133] Mitchell A.K., Bliss R.R., Church F.C. (2024). Exercise, Neuroprotective Exerkines, and Parkinson’s Disease: A Narrative Review. Biomolecules.

[134] Qiu R., Sun W., Su Y., Sun Z., Fan K., Liang Y., Lin X., Zhang Y. (2024). Irisin’s emerging role in Parkinson’s disease research: A review from molecular mechanisms to therapeutic prospects. Life Sci..

[135] Wen P., Sun Z., Yang D., Li J., Li Z., Zhao M., Wang D., Gou F., Wang J., Dai Y. (2025). Irisin regulates oxidative stress and mitochondrial dysfunction through the UCP2-AMPK pathway in prion diseases. Cell Death Dis..

[136] Islam M.R., Valaris S., Young M.F., Haley E.B., Luo R., Bond S.F., Mazuera S., Kitchen R.R., Caldarone B.J., Bettio L.E.B. (2021). Exercise hormone irisin is a critical regulator of cognitive function. Nat. Metab..

[137] Shahabi S., Esfarjani F., Zamani S., Rarani F.Z., Rashidi B. (2024). Evaluating the Efficacy of Irisin Injection in Mimicking the Molecular Responses Induced by Endurance Exercise in Mouse Liver Tissue. Int. J. Prev. Med..

[138] Ernst M., Folkerts A.K., Gollan R., Lieker E., Caro-Valenzuela J., Adams A., Cryns N., Monsef I., Dresen A., Roheger M. (2024). Physical exercise for people with Parkinson’s disease: A systematic review and network meta-analysis. Cochrane Database Syst. Rev..

[139] Cancela-Carral J.M., Campo-Prieto P., Rodriguez-Fuentes G. (2024). The IntegraPark Study: An Opportunity to Facilitate High-Intensity Exercise with Immersive Virtual Reality in Parkinson’s Disease Patients. J. Funct. Morphol. Kinesiol..

[140] Skrzatek A., Nuic D., Cherif S., Beranger B., Gallea C., Bardinet E., Welter M.L. (2024). Brain modulation after exergaming training in advanced forms of Parkinson’s disease: A randomized controlled study. J. Neuroeng. Rehabil..

[141] Hardeman L.E.S., Geerse D.J., Hoogendoorn E.M., Nonnekes J., Roerdink M. (2024). Remotely prescribed, monitored, and tailored home-based gait-and-balance exergaming using augmented reality glasses: A clinical feasibility study in people with Parkinson’s disease. Front. Neurol..

[142] Zhang T., Liu W., Bai Q., Gao S. (2023). The therapeutic effects of yoga in people with Parkinson’s disease: A mini-review. Ann. Med..

[143] He S., Ru Q., Chen L., Xu G., Wu Y. (2024). Advances in animal models of Parkinson’s disease. Brain Res. Bull..

